# Paradoxes of the EphB1 receptor in malignant brain tumors

**DOI:** 10.1186/s12935-017-0384-z

**Published:** 2017-02-08

**Authors:** Wenqiang Wei, Hongju Wang, Shaoping Ji

**Affiliations:** 1 Laboratory of Cell Signal Transduction, Medical School, Henan University, Kaifeng, 475004 China; 2 Department of Microbiology, Medical School, Henan University, Kaifeng, 475004 China; 3 Department of Anatomy, Medical School, Henan University, Kaifeng, 475004 China; 4 Department of Oncology, The First Affiliated Hospital, Henan University, Kaifeng, 475001 China

**Keywords:** EphB1, Glioma, Medulloblastoma

## Abstract

Eph receptors are a subfamily of receptor tyrosine kinases. Eph receptor-mediated forward and ephrin ligand-mediated reverse signalings are termed bidirectional signaling. Increasing evidence shows that Eph/ephrin signaling regulates cell migration, adhesion, morphological changes, differentiation, proliferation and survival through cell–cell communication. Some recent studies have started to implicate Eph/ephrin signaling in tumorigenesis, metastasis, and angiogenesis. Previous studies have shown that EphB1 receptor and its ephrin ligands are expressed in the central nervous system. EphB1/ephrin signaling plays an important role in the regulation of synapse formation and maturation, migration of neural progenitors, establishment of tissue patterns, and the development of immune organs. Besides, various recent studies have detected the abnormal expression of EphB1 receptor in different brain tumors. However, the underlying molecular mechanisms of EphB1/ephrins signaling in the development of these tumors are not fully understood. This review focuses on EphB1 that has both tumor-suppressing and -promoting roles in some brain tumors. Understanding the intracellular mechanisms of EphB1 in tumorigenesis and metastasis of brain tumors might provide a foundation for the development of EphB1-targeted therapies.

## Background

Erythropoietin-producing hepatocellular carcinoma (Eph) receptors are a subfamily of receptor tyrosine kinases (RTKs) [1, 2]. Each Eph receptor includes a kinase domain, sterile alpha motif (SAM), PDZ-binding motif, juxtamembrane segment in the intracellular region, two fibronectin type III domains and an ephrin-binding domain in the extracellular region [3, 4]. In the human genome, the Eph receptor families include ten EphA receptors and six EphB receptors [4]. The structural difference between EphA and EphB receptors is that the former promiscuously binds glycosylphosphatidylinositol-linked ephrin-A ligands, whereas the latter promiscuously binds transmembrane ephrin-B ligands [3].

**Fig. 1:**
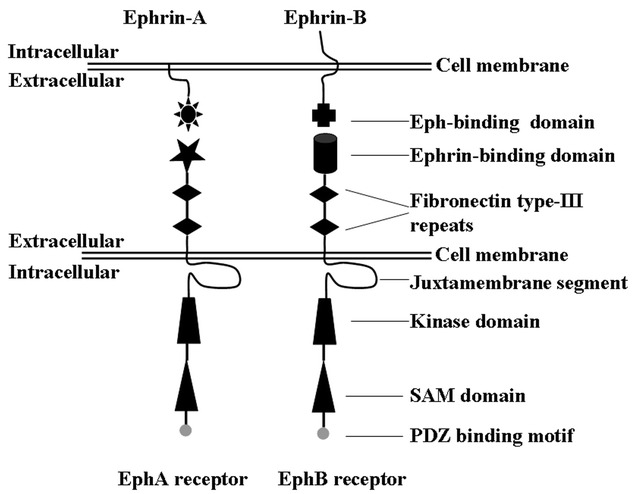
Domain structure of Eph receptors and ephrins. *SAM* sterile alpha motif, *PSD95* PDZ-postsynaptic density 95, *Dlg* discs large, *ZO-1* zonula occludens-1

**Fig. 2:**
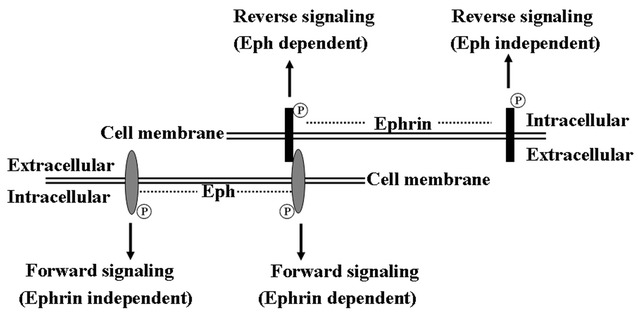
Schematic representation of Eph/ephrin bidirectional signaling. Eph receptors and ephrin ligands signal dependently or independently of each other

One of the distinctive characteristics of Eph/ephrin signaling is its ability to initiate both Eph-mediated forward and ephrin-mediated reverse signaling, termed bidirectional signaling [2]. Bidirectional propagation of signals requires the interaction between Eph receptors and ephrin ligands at sites of cell–cell contact because both are expressed at the cell surface [4] (Fig. 1). Eph-mediated forward and ephrin-mediated reverse signaling are dependent on the kinase activity of Eph receptors and ephrin ligands, respectively [5] (Fig. 2). In detail, Eph/ephrin forward signaling is initiated by ephrin ligand binding followed by the activation of tyrosine kinase domain of Eph receptor, and propagates in the Eph receptor-expressing cells while Eph/ephrin reverse signaling is triggered by Eph receptor binding followed by the activation of Src family kinase domain of ephrin ligand, and propagates in the ephrin ligand-expressing cells [6]. It should be noted that Eph receptors and ephrin ligands also signal independently of each other (Fig. 2). Nevertheless, in addition to ephrin/Eph interactions, other RTKs and adhesion molecules are involved in ligand-dependent or-independent crosstalk [4].

Eph receptors and ephrin ligands are expressed in most of cell and tissue types, and cell–cell communication mediated by Eph/ephrin signaling is implicated in various cellular behaviors, such as cell migration, adhesion, morphological changes, differentiation, proliferation, and survival [3]. It has been best characterized that Eph/ephrin signaling is involved in axonal guidance, positioning of cell populations, formation of synaptic connections and neuronal connections [3]. Recent works have begun to address the functions of Eph/ephrin signaling in vasculogenesis, bone remodeling, immune modulation and stem cell differentiation. Although considerable progress has made in our understanding of the functions of Eph/ephrin signaling in a variety of developmental processes, its roles in cancer metastasis, invasion and angiogenesis remain elusive [7]. In this review, we focus on the roles of EphB1/ephrins signaling in malignant brain tumors. More detailed information on the roles of EphA and other EphB receptors in tumorigenicity can be found in other recent reviews [2, 3, 6–8].

## EphB1 in normal nerve tissue

Eph receptors and ephrin ligands are widely expressed in all adult organs with certain organ-specific patterns. It has been shown that both Eph receptors (B1, B2, B3) and ephrin ligands (B1, B2, B3) are expressed in the central nervous system such as the adult olfactory bulb, hippocampus, and cerebellum [9]. Real-time reverse transcription polymerase chain reaction (RT-PCR) analysis has indicated that EphB1, EphA6, and EphA8 are most prominent in the brain and testis [10]. Increasing evidences have demonstrated the involvement of ephrin ligands and Eph receptors in the regulation of synapse formation and maturation, migration of neural progenitors, topographic maps, establishment of tissue patterns, and plasticity in distinct regions of the developing brain [11].

### Development and maturation of synapses

Over the last few years, the functions of Eph receptors and ephrins have been delineated in the development and maturation of synapses [12] (Table 1). It has been reported that EphB1, EphB2, and EphB3 are involved in dendritic spine morphogenesis and synapse formation in the hippocampus. In particular, EphB1 and EphB2 play the major roles in this process [13]. EphB2/ephrin-B2 forward signaling induces phosphorylation, ubiquitination and degradation of Ephexin-5, which has been shown to negatively regulate excitatory synapse development [14]. Furthermore, Eph receptors and ephrin ligands also play an important role in contact-dependent neuron–astrocyte communication at synapses [15].

**Table 1:** Summary of EphB1/ephrins functions in part of tissues and cell lines

Tissue and cell	Tissue and cell type	Expression	EphB1 functions	Ligand-dependent	Refs.
CNS^a^	Normal CNS tissue	Positive	Involved in synapse formation in the hippocampus	Dependent	[13]
CNS	Normal CNS tissue	Positive	Involved in neurogenesis of neural progenitors	Dependent	[16]
CNS	Normal CNS tissue	Positive	Involved in rerouting RGCb projections	Dependent	[17]
CNS	Normal CNS tissue	Positive	Increases expression of Nurr1	Dependent	[18–20]
P19	Embryonic carcinoma cell	Positive	Promotes cell growth and migration	Dependent	[50]
U87	Glioma cell	Undetectable	Inhibits cell migration and invasion after overexpression	Dependent	[26]
U251	Glioma cell	Undetectable	Inhibits cell migration and invasion after overexpression	Dependent	[26]
Daoy	Medulloblastoma	Overexpressed	Promotes cell growth and migration	Unknown	[48–51]
HEK 293T	Stable cell line expressing ephrin-B1	Overexpressed	Induces the activation of C-Jun N-terminal kinase activation	Dependent	[44]
U87	Glioma cells transfected with ephrin-B2	Overexpressed	Stimulates the migration and invasion	Unknown	[41]
Striatal anlage	Normal CNS tissue	Positive	Involved in the migration of two sets of neurons	Dependent	[25]
CNS	Normal CNS tissue	Positive	Regulates the proliferation and migration of neural progenitors	Dependent	[16]

^a^Represents central nervous system

^b^Represents retinal ganglion cell

### Regulation of neural progenitors

EphB1 is required for neurogenesis and migration of neural progenitors (Table 1). For instance, EphB1 and ephrin-B3 cooperatively regulate the proliferation and migration of neural progenitors in the hippocampus [16]. A lack of EphB1 significantly reduces the number of neural progenitors and nestin-positive stem cells in the hippocampus, disrupts proper migration and organization of neural progenitors, and affects other aspects of neurogenesis such as polarity, cell positioning and proliferation [16]. Furthermore, a recent study has indicated that mice lacking EphB1 and EphB2 display a positioning defect of CA3 hippocampal pyramidal neurons. EphB1 is also specifically required for rerouting retinal ganglion cell (RGC) projections ipsilaterally [17].

### Nurr1

Ligand-activated EphB1 regulates the development of the neuronal system by increased expression of Nurr1, which promotes dopaminergic neuron differentiation, neuronal survival, axonal growth arrest, and synapse formation [18–20]. Previous studies have found that EphB1 recruits Nck to stimulate the JNK pathway that promotes Nurr1 expression by binding to the AP1/c-jun binding site in the 5′-flanking region of the Nurr1 gene [21]. In addition, EphB1 receptor upregulates the expression of N-methyl-D-aspartate receptors and leads to the formation of membrane ion channels [22, 23]. Ion channels are permeable to Ca^2+^ that binds to the cAMP response element (CRE)-binding site on Nurr1 and stimulates its expression [24].

### EphB1 ligands

Ephrin-B1, ephrin-B2 and ephrin-B3 are the major ligands recognized by the EphB1 receptor (Table 1). EphB1/ephrins reverse signaling has a distinctive effect on neurons produced at the same time and site. As an activator, it can act as a repulsive signal for migrating cortical neurons [25]. However, EphB1/ephrins reverse signaling can also inhibit the migration of striatal neurons [25]. The difference in the downstream molecular machinery of neurons may contribute to the different physiological responses of the same ligand/receptor combination [25].

## EphB1 in immune system

Eph/ephrin signaling is implicated in the development of immune organs. For example, ephrin-B1 and ephrin-B2 can stimulate T cells and regulate thymocyte development [26, 27]. Further study found that the deletion of ephrin-B1 and/or ephrin-B2 in thymocytes or thymic epithelial cells (TECs) leads to a decrease of medullary areas and an enlargement of cysts [28]. However, the knockout of ephrin-B1 or ephrin-B2 in mice has no influence on the activation and proliferation of T cells and native CD_4_ cells, suggesting that other members of ephrin family may compensate the function of ephrins [26, 27]. Moreover, EphB2 and its ligands, ephrin-B1 and ephrin-B2 play a role in T cell progenitor migration [29]. Furthermore, EphB2 and EphB3 are required for the survival of the TECs and their absence will lead to a decrease of thymic cells and a reduction in volume of gland [30].

The knowledge about the role of EphB1/ephrin in immune system is limited. Previous study found that ephrin-B1 is highly expressed in peripheral blood lymphocytes (PBLs) derived from patients with rheumatoid arthritis (RA) [31]. Ephrin-B1 and EphB1 play an important role in the inflammatory condition of RA through influencing the function of T cells [31]. For instance, ephrin-B1 can activate EphB1 and stimulate the production of TNF-alpha in PBLs and IL-6 in synovial cells [31]. The function of EphB1/ephrin signaling in the development of immune organs and the corresponding mechanism of immune regulation are worth studying in the future. It is also promising to investigate whether the immune response initiated by EphB1/ephrin signaling is involved in tumorigenicity of brain tumors.

## Tumor-suppressing roles of EphB1

EphB1/ephrins signaling has perplexing dichotomous roles with tumor-suppressing and -promoting functions depending on the cellular context (Table 1).

### EphB1 suppresses glioma motility

The functions of EphB1/ephrins signaling in glioma are now beginning to be uncovered. Using large-scale data from the International Cancer Genome Consortium (ICGC) (https://dcc.icgc.org), it can be found that EphB1 alterations were observed in 4 of 268 glioma samples (1.49%), including 2 synonymous mutations and 2 missense changes. An in vivo analysis showed that the mRNA level of EphB1 expression did not vary across different glial tumor grades except for the increased expression level of EphB1 in oligodendroglioma compared with normal brain specimens. Moreover, survival analysis indicated that glioblastoma multiforme (GBM) patients with higher EphB1 expression level show longer survival rates [32]. It is proposed that ligand-dependent EphB1 signaling appears to serve as a negative regulator of glioma cell motility, and its high expression is a positive predictor for survival of GBM patients [32].

**Fig. 3:**
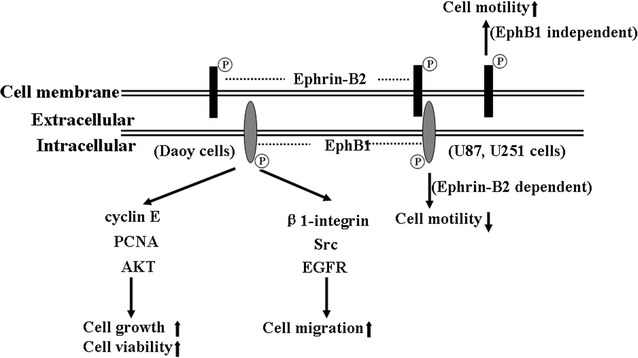
Putative model of the functions of EphB1/ephrins signaling in brain tumors. EphB1 kinase has three signaling pathways. EphB1 is phosphorylated upon stimulation by ephrin-B2. This forward signaling suppresses the motility of glioma cell lines U87 and U251. However, it also promotes the migration of medulloblastoma cell line DAOY via an unknown mechanism. Ephrin-B2 reverse signaling induces glioma cell migration independent of EphB1 binding. *P* tyrosine phosphorylation

Quantitative real-time PCR and western blotting assays indicated that U87 and U251 glioma cells display low levels of mRNA and undetectable protein of EphB1 [32]. Migration and invasion assays showed that forced overexpression of EphB1 in U251 cells effectively inhibits the cell migration and invasion upon ephrin-B2 ligand stimulation. These results indicated that enhanced EphB1 forward signaling decreases the migration and invasion of glioma, which may be associated with the survival of GBM patients [32] (Fig. 3). This is similar to the roles of EphB4. Previous studies showed that overexpression of EphB4 involves in tumor progression by promoting angiogenesis, increasing survival, and promoting invasion and migration [33, 34]. However, these effects can be inhibited in the presence of ephrin-B2 ligand, suggesting that ligand-dependent EphB4 has the tumor suppression role [33, 34]. Furthermore, EphB1 inhibits ephrin-B2 induced migration and invasion in U87 and U251 cells. However, the underlying action mechanism of EphB1 in glioma remains elusive.

### Hypermethylation

A reduction in EphB1 expression level was detected in a wide array of types of tumors such as glioma, gastric cancer, colorectal cancer, ovary serous carcinoma, and renal cell carcinoma [32, 35–38]. The mechanism that causes the decrease of EphB1 expression in these tumors remains unclear. One possible reason may be due to alternation of hypermethylation in CpG islands located in EphB1 promoter. The alternation of hypermethylation in promoter regions can influence gene expression levels in both normal and tumor cells. There is evidence for direct regulation of Eph receptors and ephrin ligands expression by methylation modifications. For example, hypermethylation of the promoter regions of almost all Eph receptors and ephrin ligands, including EphB1 and ephrin-B2, has been found in acute lymphoblastic leukemia bone marrow samples and cell lines [39]. A recent study demonstrated an inverse correlation between the expression of EphB1 transcripts and EphB1 promoter methylation in pediatric acute myelogenous leukemia [40].

### Ephrin–Eph complexes

Another possible reason for the decrease of EphB1 expression in some tumors may be that ephrin–Eph complexes can be processed by various mechanisms. The first mechanism involves protease-mediated degradation of ephrin–Eph complexes. A recent study indicated that phosphatase with tensin homology (PTEN) not only impairs EphB1-dependent cell attachment and migration, but also releases tyrosine phosphatases and ubiquitin ligase Cbl to degrade EphB1 [41]. This finding is consistent with previous studies that found that Cbl can be recruited to ephrin–Eph complexes and then dephosphorylate and degrade the receptor proteins, resulting in termination of Eph/ephrin signaling [41–44]. Moreover, membrane-anchored membrane type-1 matrix metalloproteinase (MT1-MMP) has been found to cleave EphA2 and trigger intracellular EphA2 translocation, leading to increased RhoA activity, cell junction disassembly, and single cancer cell invasion via cell repulsion [45]. It is unknown whether MT1-MMP also has certain effect on the EphB1 receptor. Another mechanism involves endocytosis mediated by cell membrane-derived vesicles, which leads to the removal of ephrin–Eph complexes from the cell surface [46].

### Ephrin-B2 reverse signaling in glioma

Interestingly, ephrin-B2 reverse signaling triggers dramatic morphological changes and increases cell motility [32, 47]. Microarray technology and immunohistochemistry assay indicated that level of ephrin-B2 mRNA is significantly higher in GBM than in normal brain, and associated with short-term survival in malignant astrocytomas. Forced expression of ephrin-B2 stimulates the migration and invasion in the U251 cells [48]. However, EphB1 partially abrogates the migration and invasion induced by ephrin-B2 reverse signaling in glioma cells [32]. It is unclear which molecules are implicated in the phenotypic changes of glioma cells downstream of ephrin-B2 and how EphB1-ephrin-B2 interactions cooperatively regulate the cellular behavior of glioma cells.

A previous study has shown that ephrin-B2 controls cell motility and adhesion during blood vessel wall assembly by EphB4-ephrin-B2 interactions that recruit the adaptor molecule Crk and p130 (Cas) signaling complex to reduce cell motility [49]. Cho et al. found that activation of ephrin-B1 by EphB1/Fc can lead to JNK activation, which is a downstream event of ephrin-B reverse signaling. A further study showed that ephrin-B1 interacts with CNK1 and promotes cell migration through RhoA-dependent JNK activation [50]. Recent study demonstrated that ephrin-B2 control vessel pruning through STAT1–JNK3 signalling and plays a important role in the endothelial cell survival [51]. The Src kinase could positively regulate ephrinB phosphorylation whereas tyrosine phosphatase PTB-BL could de-phosphorylate ephrin-B2 [52]. The signals inducing ephrin-B2 de-phosphorylation will lead to endothelial cell death. Future studies will be needed to determine whether EphB1 interacts with these phosphatases and kinases to regulate the ephrin-B2 reverse signaling. In addition, it has been proposed that the feedback loop mechanism of Eph/ephrin signaling exists in glioma cells. For instance, ephrin-A1 suppresses EphA2 expression and vice versa, thus regulating cell migration and invasion [1]. Ephrin-B1 also decreases EphB2 expression levels by inducing EphB2 internalization and degradation [6, 53]. It also needs to be determined whether the feedback between EphB1 and ephrin exists in glioma cells.

By now, the direct evidences that clarify the mechanism of EphB1 in inhibiting glioma cell migration and invasion are limited. We are now designing experiments to determine the detailed action mechanism of EphB1 in inhibiting the migration of glioma cells.

## Tumor-promoting roles of EphB1

In addition to the tumor-suppressing roles of EphB1 in diverse tumor types, it controversially has tumor-promoting roles. The different roles of EphB1 in tumor development can be partially explained by the fact that the function of Eph receptors is highly context-dependent and can vary across cancer types [6].

### EphB1 promotes tumorigenesis in medulloblastoma

In addition to the tumor-suppressing role in glioma, EphB1 also promotes tumorigenesis in medulloblastoma [54]. A recent study found that mRNA and protein expression levels of EphB1 are high in human medulloblastoma cell lines Daoy and UW228. EphB1 knockdown in Daoy using siRNA assay reduces cell growth and migration, decreases the expression of important cell cycle regulators, and increases the percentage of cells in G1 phase of the cell cycle [54, 55]. These changes in cell physiology due to EphB1 knockdown can be explained in part by the decrease in the expression levels of cyclin E, PCNA, and AKT [54] (Fig. 3). Moreover, EphB1 knockdown decreases β1-integrin expression and phosphorylated Src levels, which is consistent with a previous study indicating that EphB1 regulates cell migration and chemotaxis via stimulation of c-Src activity [54, 56] (Fig. 3). Furthermore, EphB1 can functionally interact with the epidermal growth factor receptor (EGFR), contributing to the metastatic behavior of medulloblastoma cells [54, 57]. Importantly, EphB1 knockdown can enhance the sensitivity of medulloblastoma to ionizing radiation sensitivity in vitro and in vivo [54]. It should be noted that IDH mutation status of diffuse gliomas is required for proper subclassification [58]. Up to now, studies of Ephrin/Eph have not determined whether tumor biology differs according to IDH status.

### Tumor-promoting mechanisms

Previous studies utilized some cell lines, such as P19 cells, human renal microvascular endothelial cells, and CHO cells, to explore the action mechanism of EphB1 in cell migration and invasion. Increasing evidence suggests that the EphB1 and ephrin complex functions in cooperation with other signaling molecules to regulate cellular behaviors.

### Grb2

**Fig. 4:**
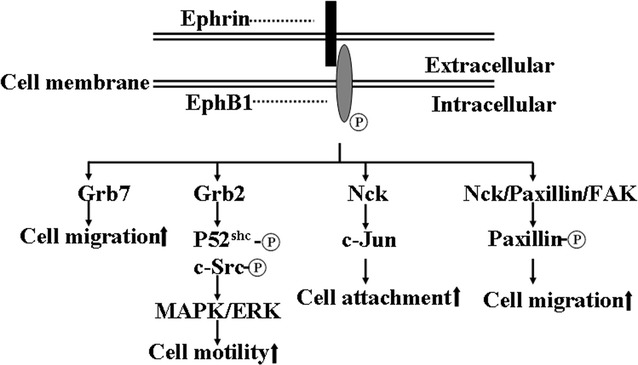
Cellular proteins interacting with EphB1. Ligand-activated EphB1 recruits adaptor proteins Grb2, Grb7, Nck and paxillin, and is involved in cell migration and adhesion

It has been determined that EphB1 recruits adaptor proteins and promotes cell migration (Fig. 4). For instance, activated EphB1 recruits adaptor protein Grb2 and p52^Shc^, and promotes phosphorylation of p52^Shc^ and c-Src, whereby their concerted actions activate MAPK/ERK and regulate events involved in cell motility (Fig. 4) [56]. Grb2 has been known to be associated with tyrosine phosphorylated EGFRs and platelet-derived growth factors (PDGFRs) through its Src homology 2 (SH2) domain and couples the receptor tyrosine kinase to Ras signaling [59].

### Grb7

In addition to Grb2, the interaction between EphB1 and adaptor protein Grb7 plays a role in the regulation of cell migration (Fig. 4) [60]. SH2 domain of Grb7 can interact with FAK, and involve in intergrin-mediated cell migration [61, 62]. Interestingly, FAK also recruits many signaling molecules, such as Grb2, Grb7, Src and PI3K, to local contacts and stimulate the cell migration (Fig. 4) [63].

### Signaling complex

Ligand-activated EphB1 forms a signaling complex with Nck, paxillin, and FAK, and induces tyrosine phosphorylation of paxillin in a c-Src-dependent manner (Fig. 4) [64]. The biological function of paxillin coordinated signaling may involve in the regulation of cell spreading and motility [65]. Taken together, these data suggest that Grb2, Grb7, integrins, Nck, NIK, Src, paxillin and other adaptor proteins play essential roles in cell migration mediated by EphB1/ephrins signaling.

These accumulated data have lead to a better understanding of the roles of EphB1 in cell motility. More studies are still required to comprehensively elucidate the crosstalk between EphB1 with these signaling molecules for understanding the action mechanism of EphB1 on the migration and invasion of tumor cells.

## Tumor microenvironment

Tumor microenvironment is an emerging research field of tumor biology in recent years. It is mainly characterized by hypoxia, angiogenesis, lower extracellular pH in tumor tissues [66]. The roles of EphB1/ephrin signaling in tumor microenvironment are less known by now.

### Hypoxia

Previous studies have shown that the mRNA and protein expression levels of hypoxia inducible factor (HIF)-1 alpha, vascular endothelial growth factor (VEGF), EphB4, ephrin-B2, EphA2 and ephrin-A1 are increased in the skin upon of induction of hypoxia while knockdown of HIF-1alpha leads to an abolishment of up-regulation of Eph receptors and ephrin ligands [67, 68]. However, another study observed that hypoxia has no effect on the Eph/ephrin expression in human melanoma cells in vitro [69]. The relationship between the protein expression of Eph/ephrin and hypoxia condition remains elusive. Moreover, it has been known that dysregulation of ephrin/Eph may influence the cell–matrix and cell–cell attachment, and organization of the cytoskeleton, which can increase tumor cell invasion and metastasis. For instance, ephrin ligand and Eph receptor may interact with adhesion molecule E-cadherin and affect cell–cell attachment [70, 71].

### Angiogenesis

Eph/ephrin signaling plays a key role in the angiogenesis of some tumors. Tumor angiogenesis plays an important role in the metastasis and carcinogenesis. Previous study have found that ephrin-B2 reverse signaling is implicated in endothelial cell sprout and tip cell generation and elongation [72]. It has also been determined that EphB4 and ephrin-B2 mRNAs were expressed at sites of neovascularization of human glioma while the ephrin-B1 was only detected in the proximity of vessels, suggesting the functional interaction between Eph receptors and ephrin ligands expressed on endothelial and glioma cells, respectively [73]. Moreover, the truncated and soluble EphB4 receptor prompts tumor angiogenesis upon stimulating ephrin-B2 signaling [48]. In contrast, soluble ephrin-B2-Fc molecule suppresses growth of head and neck squamous cell carcinoma xenografts by inducing maturation/stabilization of vessels in the tumor [74]. Furthermore, ephrin-B2 is involved in the angiogenesis induced by VEGF signaling pathway by facilitating VEGF receptor internalization [75].

## EphB1 receptor as a potential therapeutic target in brain tumors

Certain molecules have been developed to specifically target Eph/ephrin bidirectional signals, such as antagonistic antibodies, peptides, and recombinant proteins [76–79]. The high expression level of EphB1 in part of brain tumors, like medulloblastoma, has raised interest in exploring new strategies to target EphB1 receptors for cancer therapy. For instance, the molecules to inhibit EphB1 kinase signaling, such as ligand-blocking antibodies and peptides, can be used as antagonists [76, 77, 80]. Moreover, radiation therapy could be used in combination with knockdown of EphB1 by RNA interference technique to enhance cellular radiosensitization of tumor cells [54]. Another future research direction will be the use of cytotoxic payloads delivered by antibody–drug conjugates to interfere with EphB1/ephrins signaling for inhibiting tumor cell growth [79, 80]. It should be noted that the blood–brain barrier (BBB) might influence the availability of the biologic drugs in the brain. Therefore, the new methods should be developed to cross the BBB. For instance, drugs can bypass the BBB by conjugating with a monoclonal antibody against the transferrin receptor, injecting the effective cell-penetrating peptides, or using focused ultrasound and circulating microbubbles [81–83]. Further in vitro and in vivo studies are still needed to determine whether these approaches are effective treatments for some brain tumors.

## Conclusions

The action mechanism of EphB1/ephrins signaling appears to be complex in normal central nervous tissue and brain tumors. EphB1/ephrins signaling is not only implicated in suppressing tumor migration and invasion, but also promoting tumor development. By now, our understanding on the complicated functions of EphB1/ephrins signaling in brain tumors is still limited, and more studies are urgently needed to resolve the confusing and controversial events. First, appropriate studies are crucial to decipher the paradoxes of EphB1/ephrins signaling in different cellular contexts during the development of a variety of brain tumors. In particular, it is worth exploring the unknown molecular mechanisms of EphB1, such as genetic and epigenetic modifications, downstream signaling pathways, the feedback loop between the ligands and receptors, and the protein degradation process of EphB1, to expand our knowledge about the molecular pathogenesis of brain tumors [32]. Moreover, it will be important to investigate whether EphB1 mutations widely exist in brain tumor patients and the effect of these mutations on tumorigenesis. Recent studies have found gene mutations in the kinase domain of EphA3 in colorectal cancer cells [4]. Furthermore, it is essential to explore the correlation of EphB1 expression levels with the clinical outcomes of various brain tumor types in order to determine whether EphB1 is a favorable prognostic predictor for patients. In conclusion, the roles of EphB1/ephrins signaling in brain tumors are only beginning to be explored, and further studies will generate more comprehensive data.

## Abbreviations

Eph: erythropoietin-producing hepatocellular carcinoma
RTK: receptor tyrosine kinase
GBM: glioblastoma multiforme
SAM: sterile alpha motif
CRE: cAMP response element
PP2A: protein phosphatase 2A
RT-PCR: real-time reverse transcription polymerase chain reaction
PTEN: phosphatase with tensin homology
MT1-MMP: membrane-anchored membrane type-1 matrix metalloproteinase
FAK: focal adhesion kinase
EGFR: epidermal growth factor receptor
PDGFR: platelet-derived growth factors
SH2: Src homology 2
